# Supplementary material for: Toxicity, Pharmacokinetics, and Gut Microbiome of Oral Administration of Sesterterpene MHO7 Derived from a Marine Fungus
Source: Mar Drugs. 2019 Nov 26;17(12):667. doi: 10.3390/md17120667 (PMC6950057; doi:10.3390/md17120667)
Supplement: Supplementary file 1 [file marinedrugs-17-00667-s001.zip › supplementary materials-1-clean file.pdf]

Toxicity, Pharmacokinetics, and Gut microbiome of Oral Administration of Sesterterpene MHO7 Derived from a Marine Fungus

Wei Tian, Liang Yang, Di Wu, Zixin Deng and Kui Hong *

Key Laboratory of Combinatorial Biosynthesis and Drug Discovery, Ministry of Education, and Wuhan University School of Pharmaceutical Sciences, Wuhan University, Wuhan 430071, China; twwtss@163.com (W.T.); liangy@whu.edu.cn (L.Y.); diwu0301@163.com (D.W.); zxdeng@whu.edu.cn (Z.D.)

* Correspondence: kuihong31@whu.edu.cn; Tel.: +86-27-6875-2442

Received: date; Accepted: date Published: date

Supplementary Information

Table S1. Intra-day and inter-day precision and accuracy of HPLC method
Table S2. Log P and Log D values of MHO7 in different time and pH condition at 37 ℃.
Table S3. Log P value of MHO7 in different time at 25 ℃.
Table S4. Method validation of MHO7 for *in vitro* incubation system
Table S5. Weight of mice organs in acute toxicity test
Table S6. Method validation of MHO7 in plasma
Table S7. Method validation of MHO7 in tissues
Table S8. The concentration of MHO7 in mice tissues
Table S9. The tissue-to-plasma radio of MHO7 in different time
Table S10. Significant different phyla between control and treatment groups
Table S11. Significant different classes between control and treatment groups
Table S12. Significant different orders between control and treatment groups
Table S13. Significant different families between control and treatment groups
Table S14. Significant different genera between control and treatment groups
Figure S1. Specificity of MHO7 *in vitro* incubation system
Figure S2. Body weight changes of female and male mice within 14 days
Figure S3. Specificity of MHO7 in plasma
Figure S4. Specificity of MHO7 in tissues
Figure S5. Rarefaction and Shannon index curves of OTU level
Figure S6. LDA score by Linear Discriminant Analysis (LDA) Effect Size (LefSe) analysis
Figure S7. MHO7 modulated the composition of gut microbiota at the different taxonomic levels

Table S1. Intra-day and inter-day precision and accuracy of HPLC method

Concentration (mM)	Inter-day Precision (RSD, %)	Intra-day Precision (RSD, %)	Accuracy (%)
1	0.17	0.10	101.01±0.10
0.1	0.21	1.33	100.48±1.33
0.01	0.87	0.76	99.88±0.76

Table S2. Log P and Log D values of MHO7 in different time and pH condition at 37 °C.

Time (h)	Log P/D Value			
	O/P (pH 1.5)	O/P (pH 5.0)	O/P (pH 7.4)	O/W (pH 7.0)
4	1.29 ±0.02	1.15 ±0.02	1.39 ±0.03	2.55 ±0.01
8	1.22 ±0.04	1.03 ±0.05	1.37 ±0.05	2.51 ±0.04
12	1.13 ±0.03	0.95 ±0.03	1.31 ±0.03	2.48 ±0.02
24	1.13 ±0.02	0.95 ±0.04	1.29 ±0.02	2.49 ±0.02
30	1.12 ±0.15	0.95 ±0.03	1.27 ±0.04	2.47 ±0.01
36	1.12 ±0.03	0.93 ±0.03	1.26 ±0.05	2.49 ±0.03
48	1.12 ±0.03	0.93 ±0.02	1.26 ±0.01	2.48 ±0.03

O, means octanol; P, means PBS; W, means ddH_2O.

Table S3. Log P value of MHO7 in different time at 25 °C.

Time (h)	Log P Value
4	1.23 ±0.02
8	1.22 ±0.04
12	1.23 ±0.03
24	1.23 ±0.02
30	1.27 ±0.15
36	1.27 ±0.03
48	1.29 ±0.05

Table S4. Method validation of MHO7 *in vitro* incubation system

Concentration (mM)	HQC	MQC	LQC
Inter-day Precision (RSD, %)	4.97	3.34	7.36
Intra-day Precision (RSD, %)	6.42	7.24	7.95
Inter-day Accuracy (%)	97.35	98.66	101.86
Intra-day Accuracy (%)	96.42	95.70	97.26
Matrix effect (%, mean ± SD)	91.45 ± 3.23	95.77 ± 2.98	95.24 ± 4.31
Recovery (%, mean ± SD)	98.99 ± 1.08	99.24 ± 2.02	97.65 ± 1.36
1 freeze-thaw cycles Precision (RSD, %)	2.22	2.15	3.53
2 freeze-thaw cycles Precision (RSD, %)	2.36	2.89	3.77
3 freeze-thaw cycles Precision (RSD, %)	5.87	2.56	4.44
1 freeze-thaw cycles Accuracy (%)	98.87	101.86	103.65
2 freeze-thaw cycles Accuracy (%)	99.56	101.33	101.00
3 freeze-thaw cycles Accuracy (%)	97.24	96.99	98.28
6 h – stability Precision (RSD, %)	2.48	3.71	4.76
12 h – stability Precision (RSD, %)	3.01	3.22	4.06
24 h – stability Precision (RSD, %)	3.53	3.85	5.36
48 h – stability Precision (RSD, %)	3.24	3.60	6.89
6 h – stability Accuracy (%)	101.38	101.26	98.36
12 h – stability Accuracy (%)	101.99	100.83	97.25
24 h – stability Accuracy (%)	102.03	99.10	98.62
48 h – stability Accuracy (%)	101.63	99.25	97.47
24 h –automatic sampler stability Precision (RSD, %)	3.85	4.10	4.44
48 h –automatic sampler stability Precision (RSD, %)	5.77	8.92	7.17
24 h –automatic sampler stability Accuracy (%)	99.69	97.01	98.34
48 h –automatic sampler stability Accuracy (%)	100.64	98.12	95.91

The calibration curves was Y = 0.0303851+1.4835*X, R^2 = 0.9960. The lower limit of quantitation of MHO7 was 0.01 $\mu M \cdot L^{-1}$.

Table S5. Weight of mice organs in acute toxicity test

Tissues	♀		♂	
	Control	2400 mg/kg w	Control	2400 mg/kg w
Body weight	33.53±2.80	33.30±2.1	41.38±2.60	38.75±2.96
Heart	0.16±0.01	0.15±0.01	0.18±0.01	0.18±0.02
Liver	1.44±0.17	1.49±0.09	2.40±0.23	2.39±0.38
spleen	0.09±0.02	0.13±0.05	0.10±0.03	0.15±0.03
lung	0.24±0.03	0.22±0.02	0.23±0.04	0.22±0.03
kidney	0.41±0.04	0.46±0.02	0.60±0.04	0.58±0.06
Reproductive organs	0.32±0.12	0.21±0.05	0.21±0.02	0.19±0.02
brain	0.43±0.02	0.40±0.07	0.43±0.04	0.42±0.06

Table S6. Method validation of MHO7 in plasma

Concentration (mM)	HQC	MQC	LQC
Inter-day Precision (RSD, %)	2.02	2.39	3.77
Intra-day Precision (RSD, %)	4.23	5.89	5.47
Inter-day Accuracy (%)	97.19	96.32	93.57
Intra-day Accuracy (%)	94.48	92.50	94.31
Matrix effect (%, mean ± SD)	100.63 ± 3.84	93.13 ± 5.95	90.57 ± 4.11
Recovery (%, mean ± SD)	90.09 ± 3.37	90.61 ± 5.01	90.27 ± 3.92
1 freeze-thaw cycles Precision (RSD, %)	1.22	2.76	1.70
2 freeze-thaw cycles Precision (RSD, %)	2.34	3.58	4.20
3 freeze-thaw cycles Precision (RSD, %)	3.35	4.97	3.38
1 freeze-thaw cycles Accuracy (%)	96.53	96.21	93.90
2 freeze-thaw cycles Accuracy (%)	97.41	96.30	93.57
3 freeze-thaw cycles Accuracy (%)	97.36	95.14	93.80
6 h – stability Precision (RSD, %)	3.89	3.10	4.52
12 h – stability Precision (RSD, %)	5.18	4.88	3.74
24 h – stability Precision (RSD, %)	4.27	4.86	6.58
48 h – stability Precision (RSD, %)	4.07	3.53	5.72
6 h – stability Accuracy (%)	94.45	92.83	92.75
12 h – stability Accuracy (%)	97.35	94.46	93.31
24 h – stability Accuracy (%)	98.92	95.81	91.95
48 h – stability Accuracy (%)	95.33	96.49	94.89
7 d – stability Precision (RSD, %)	2.81	4.34	6.74
15 d – stability Precision (RSD, %)	3.44	5.38	4.80
30 d – stability Precision (RSD, %)	2.92	4.93	3.84
7 d – stability Accuracy (%)	94.02	94.94	92.80
15 d – stability Accuracy (%)	92.48	91.18	93.51
30 d – stability Accuracy (%)	93.39	94.63	94.75
24 h –automatic sampler stability Precision (RSD, %)	5.41	4.35	5.43
48 h –automatic sampler stability Precision (RSD, %)	5.73	5.68	5.94
24 h –automatic sampler stability Accuracy (%)	93.01	95.36	92.33
48 h –automatic sampler stability Accuracy (%)	91.59	93.83	93.76

The calibration curves was Y = 0.0486392+1.8329*X, R^2 = 0.9982. The lower limit of quantitation of MHO7 was 0.01 $\mu M \cdot L^{-1}$.

Table S7. Method validation of MHO7 in tissues

Concentration (mM)	GI-HQC	GI-MQC	GI-LQC	L-HQC	L-MQC	L-LQC
Inter-day Precision (RSD, %)	4.33	6.79	6.68	4.87	4.27	4.63
Intra-day Precision (RSD, %)	5.86	4.25	8.76	5.13	5.65	6.94
Inter-day Accuracy (%)	99.24	100.62	97.23	98.55	98.23	100.26
Intra-day Accuracy (%)	103.21	97.36	94.25	99.43	97.88	101.15
Matrix effect (%, mean ± SD)	101.37 ± 2.36	98.35 ± 3.28	96.20 ± 3.65	92.18 ± 1.58	91.42 ± 2.93	90.05 ± 2.49
Recovery (%, mean ± SD)	94.38 ± 2.79	96.45± 1.64	92.81±2.55	92.28 ± 2.42	92.09 ± 1.07	93.97 ± 3.59
1 freeze-thaw cycles Precision (RSD, %)	3.64	4.20	3.82	4.47	3.33	4.34
2 freeze-thaw cycles Precision (RSD, %)	5.38	3.29	6.76	1.39	2.20	3.85
3 freeze-thaw cycles Precision (RSD, %)	5.47	7.25	7.34	1.09	3.48	3.54
1 freeze-thaw cycles Accuracy (%)	98.37	95.25	96.11	97.19	97.30	96.64
2 freeze-thaw cycles Accuracy (%)	96.73	93.26	93.16	94.31	96.59	93.76
3 freeze-thaw cycles Accuracy (%)	95.26	92.15	91.45	95.31	94.92	94.85
6 h – stability Precision (RSD, %)	3.14	3.99	5.70	1.55	2.32	3.91
12 h – stability Precision (RSD, %)	4.28	3.70	4.62	3.25	5.26	4.78
24 h – stability Precision (RSD, %)	3.02	3.23	4.55	3.54	3.88	5.97
48 h – stability Precision (RSD, %)	4.21	3.58	5.64	3.02	2.09	4.21
6 h – stability Accuracy (%)	96.31	98.03	95.48	97.49	98.28	98.4
12 h – stability Accuracy (%)	97.87	98.11	94.71	95.11	98.84	97.20
24 h – stability Accuracy (%)	96.52	97.38	94.41	97.48	96.60	98.08
48 h – stability Accuracy (%)	97.86	97.41	95.09	95.28	94.41	97.59
7 d – stability Precision (RSD, %)	4.23	4.47	5.01	3.19	4.21	3.81
15 d – stability Precision (RSD, %)	4.73	3.06	6.99	3.27	3.41	3.85
30 d – stability Precision (RSD, %)	3.06	4.99	5.78	4.24	1.83	4.25
7 d – stability Accuracy (%)	98.89	99.41	94.57	97.41	98.87	99.47
15 d – stability Accuracy (%)	99.43	96.64	95.27	94.30	98.54	95.84
30 d – stability Accuracy (%)	99.61	95.10	94.14	93.64	96.29	95.40
24 h –automatic sampler stability Precision (RSD, %)	5.28	5.36	4.86	2.39	4.97	3.71
48 h –automatic sampler stability Precision (RSD, %)	100.11	101.93	97.52	98.55	94.97	94.26
24 h –automatic sampler stability Accuracy (%)	6.01	9.54	7.73	4.80	2.93	3.75
48 h –automatic sampler stability Accuracy (%)	94.63	95.16	96.74	95.89	95.75	94.53

GI-HQC, MQC, LQC means the QC of Gastrointestinal contents samples; L-HQC, MQC, LQC means the QC of liver samples. The calibration curves of gastrointestinal contents were Y = 0.0540328+1.38011*X, R^2 = 0.9942 and Y = 0.0018527+0.72176*X, R^2 = 0.9903 for high and low concentrations, respectively, and the lower limit of quantitation of MHO7 was 0.005 $\mu M \cdot L^{-1}$. The calibration curves of liver were Y = 0.0608319+1.39753*X, R^2 = 0.9976 and Y = 0.0082588+0.26768*X, R^2 = 0.9971. for high and low concentrations, respectively, and the lower limit of quantitation of MHO7 was 0.005 $\mu M \cdot L^{-1}$.

Table S8 The concentration of MHO7 in mice tissues (n = 6)

Tissues	Concentration in different time point (μg·g^{-1})					
	1h	4h	8h	12h	20h	30h
Heart	0.06±0.037	0.85±0.51	0.67±0.48	0.64±0.30	0.04±0.02	0.08±0.01
Liver	3.01±1.53	1.64±1.09	0.18±0.22	0.49±0.37	0.06±0.05	0.01±0.01
Spleen	0.04±0.03	0.43±0.20	0.48±0.50	0.20±0.14	0.31±0.02	0.01±0.004
Lung	0.43±0.18	1.06±0.45	0.82±0.71	2.34±1.91	0.16±0.04	0.08±0.02
Kidney	0.11±0.06	1.63±0.92	1.66±0.74	8.16±6.23	0.39±0.09	0.07±0.01
Brain	0.95±0.80	0.48±0.33	0.50±0.15	0.18±0.11	0.10±0.05	0.01±0.004
Muscle	0.31±0.22	0.97±0.48	0.64±0.37	1.42±0.80	0.17±0.10	0.02±0.003
Reproductive organs	0.88±0.68	2.29±1.80	23.90±11.33	13.69±10.29	1.46±1.23	0.36±0.46
Fat	0.74±0.41	5.20±3.47	11.15±8.87	0.74±0.70	0.34±0.25	0.18±0.16

Table S9 The tissue-to-plasma radio of MHO7 in different time (n = 6)

Tissues	Kp value in different time point (μg·g^{-1})					
	1h	4h	8h	12h	20h	30h
Heart	0.31±0.29	4.20±3.57	0.71±0.62	2.02±1.87	0.93±0.88	3.58±2.98
Liver	17.40±12.82	5.02±4.70	0.18±0.11	1.22±1.06	1.68±1.21	0.32±0.22
Spleen	0.28±0.30	1.80±1.02	0.58±0.44	1.11±0.96	8.55±6.76	0.69±0.58
Lung	2.71±1.48	4.43±2.87	0.72±0.56	6.84±4.38	4.40±2.51	3.54±2.10
Kidney	0.68±0.44	5.50±3.13	1.52±0.93	17.24±13.22	9.90±7.91	3.68±2.13
Brain	6.11±3.47	1.34±0.53	0.52±0.25	0.36±0.17	2.48±1.51	0.57±0.32
Muscle	1.95±1.01	4.04±2.72	0.54±0.36	4.20±2.44	5.54±3.65	0.99±0.55
Reproductive organs	6.05±4.35	11.86±8.16	22.58±11.24	35.45±15.81	30.45±12.78	17.62±7.50
Fat	5.44±3.59	14.03±5.33	9.25±3.69	2.33±1.15	9.80±6.01	9.00±6.96

Table S10. Significant differet phyla between control and treatment groups (n = 3)

**Species name**	**Relative abundance (%)**			
	**Control group**	**MHO7-1h group**	**MHO7-8h group**	**MHO7-30h group**
Proteobacteria	0.57±0.31	0.18±0.045	2.05±1.47	1.34±1.58
Saccharibacteria	0.44±0.42	0.47±0.28	0.35±0.61	0.0069±0.0055
Tenericutes	0.25±0.20	0.46±0.25	0.0075±0.0047	0.0013±0.0031
Deferribacteres	0.14±0.17	0.14±0.12	0.0044±0.0050	0.25±0.23

Table S11. Significant different classes between control and treatment groups (n = 3)

**Species name**	**Relative abundance (%)**			
	**Control group**	**MHO7-1h group**	**MHO7-8h group**	**MHO7-30h group**
Clostridia	51.23±18.09	38.58±11.1	28.98±20.11	47.82±20.66
Bacilli	0.77±0.54	10.08±7.69	11.49±12.09	3.25±3.05
Verrucomicrobiae	0.0069±0.013	0.0019±0.002	0.80±1.18	2.51±3.67
Mollicutes	0.25±0.20	0.46±0.25	0.0075±0.0048	0.0012±0.0031

Table S12. Significant different orders between control and treatment groups (n = 3)

Species name	Relative abundance (%)			
	Control group	MHO7-1h group	MHO7-8h group	MHO7-30h group
Clostridiales	51.26±18.06	38.54±11.15	28.89±20.03	47.86±20.61
Lactobacillales	0.76±0.53	10.10±7.65	11.34±12.02	3.26±3.04
Verrucomicrobiales	0.0069±0.012	0.0022±0.0027	0.78±1.15	2.52±3.65
Mollicutes_RF9	0.23±0.21	0.33±0.16	0.0057±0.0040	0.0014±0.0025
Anaeroplasmatales	0.018±0.032	0.12±0.11	0.00028±0.00068	0.00000
Bacillales	0.0013±0.0024	0.0000	0.071±0.067	0.00034±0.00084

Table S13. Significant differet families between control and treatment groups (n = 3)

Species name	Relative abundance (%)			
	Control group	MHO7-1h group	MHO7-8h group	MHO7-30h group
Lachnospiraceae	32.11±12.99	27.23±10.42	21.66±15.88	42.58±19.32
Bacteroidales_S24-7_group	21.90±6.85	33.99±6.67	33.65±5.47	19.34±6.97
Ruminococcaceae	17.46±6.16	10.04±1.97	6.20±4.57	4.13±1.97
Lactobacillaceae	0.75±0.54	10.05±7.69	11.34±12.11	3.21±2.99
Rikenellaceae	8.37±4.69	7.09±0.86	3.66±2.13	3.19±2.16
Verrucomicrobiaceae	0.0069±0.013	0.0019±0.0021	0.80±1.18	2.51±3.67

Table S14. Significant different genera between control and treatment groups (n = 3)

Species name	Relative abundance (%)			
	Control group	MHO7-1h group	MHO7-8h group	MHO7-30h group
g__norank_f__Bacteroidales_S24-7_group	21.9±6.84	33.99±6.67	33.65±5.47	19.34±6.97
g__norank_f__Lachnospiraceae	7.89±3.26	6.456±2.68	7.54±4.66	19.81±11.07
Lactobacillus	0.75±0.54	10.05±7.69	11.34±12.11	3.21±2.99
Rikenellaceae_RC9_gut_group	4.38±2.38	2.82±0.76	0.088±0.089	0.042±0.084
Prevotellaceae_UCG-001	0.74±0.94	0.78±0.51	4.09±3.41	1.07±1.13
Ruminococcus_1	3.24±2.87	1.53±1.04	0.38±0.32	0.071±0.15
Lachnoclostridium	0.58±0.18	0.51±0.21	0.81±0.61	2.42±2.02
Ruminococcaceae_UCG-014	1.13±0.51	2.73±0.93	0.19±0.25	0.022±0.030
Ruminiclostridium_9	1.73±0.75	0.76±0.29	0.60±0.59	0.41±0.20
Prevotellaceae_NK3B31_group	0.28±0.46	0.46±0.31	2.54±2.44	0.14±0.19
Ruminiclostridium	2.15±1.27	0.78±0.59	0.36±0.31	0.079±0.076
g__norank_f__Ruminococcaceae	1.81±1.69	0.31±0.22	0.31±0.26	0.16±0.13
Ruminiclostridium_5	0.66±0.25	0.34±0.14	0.21±0.21	0.27±0.23
[Eubacterium]_coprostanoligenes_group	0.79±0.40	0.13±0.12	0.22±0.23	0.28±0.50
Coprococcus_1	0.57±0.34	0.19±0.10	0.21±0.17	0.11±0.068
norank_o__Mollicutes_RF9	0.23±0.21	0.34±0.17	0.0075±0.0047	0.0012±0.0031
Ruminococcaceae_UCG-010	0.32±0.17	0.12±0.046	0.036±0.042	0.0062±0.0061
Ruminococcaceae_UCG-009	0.23±0.15	0.066±0.050	0.063±0.072	0.012±0.015
Ruminococcaceae_NK4A214_group	0.086±0.053	0.076±0.024	0.023±0.021	0.020±0.023
[Eubacterium]_ventriosum_group	0.024±0.019	0.13±0.089	0.012±0.020	0.022±0.041
Anaeroplasma	0.018±0.033	0.12±0.11	00	00
Family_XIII_AD3011_group	0.031±0.016	0.067±0.041	0.0069±0.0077	0.021±0.014
g__norank_f__Christensenellaceae	0.064±0.027	0.034±0.014	0.011±0.0079	0.0069±0.0087
Sutterella	0.0044±0.0073	00	0.0069±0.010	0.090±0.090
Streptococcus	0.013±0.0081	0.023±0.0050	0.029±0.016	0.0025±0.0031
Staphylococcus	0.0012±0.0031	00	0.066±0.060	00
Peptococcus	0.038±0.030	0.010±0.0061	0.0050±0.010	0.0050±0.0056
Papillibacter	0.019±0.013	0.0062±0.0066	0.0025±0.0061	0.00062±0.0015

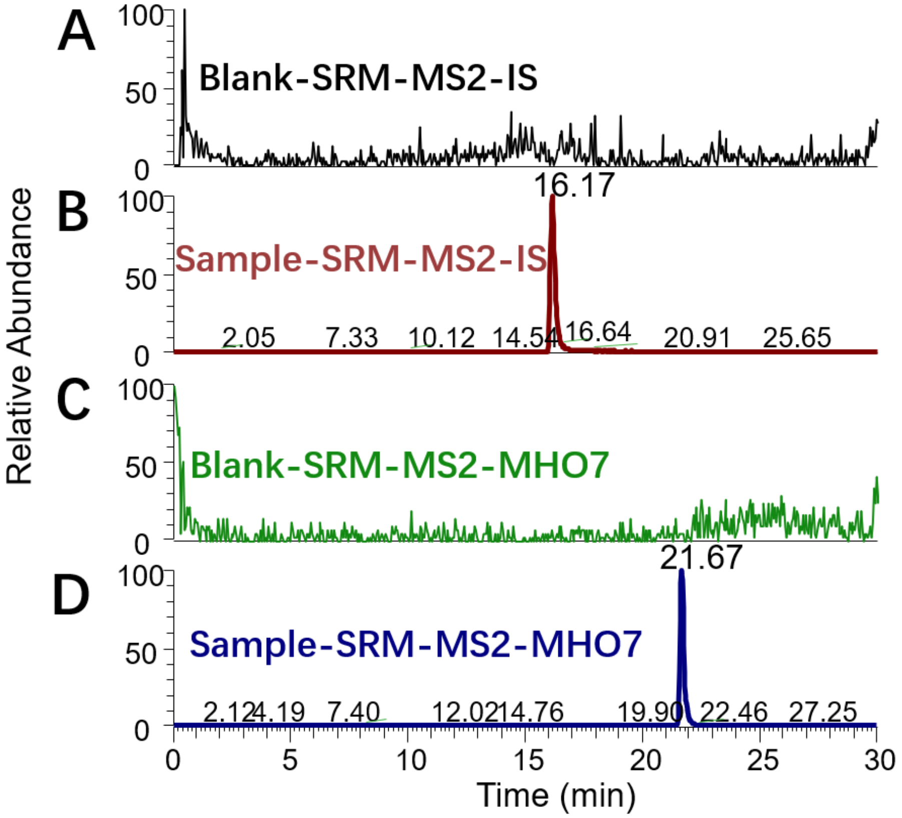

Figure S1. Specificity of MHO7 *in vitro* incubation system. (A) MS2 spectrogram of IS in blank SGF of SRM model. (B) MS2 spectrogram of IS in SGF of SRM model. (C) MS2 spectrogram of MHO7 in blank SGF of SRM model. (D) MS2 spectrogram of MHO7 in SGF of SRM model.

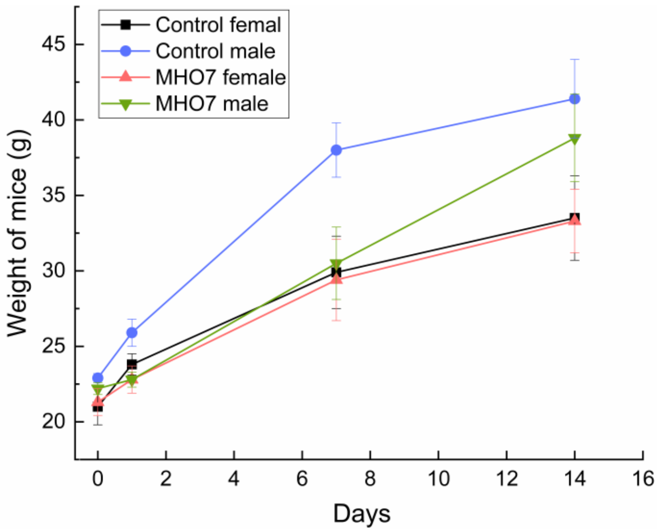

Figure S2. Body weight changes of female and male mice within 14 days

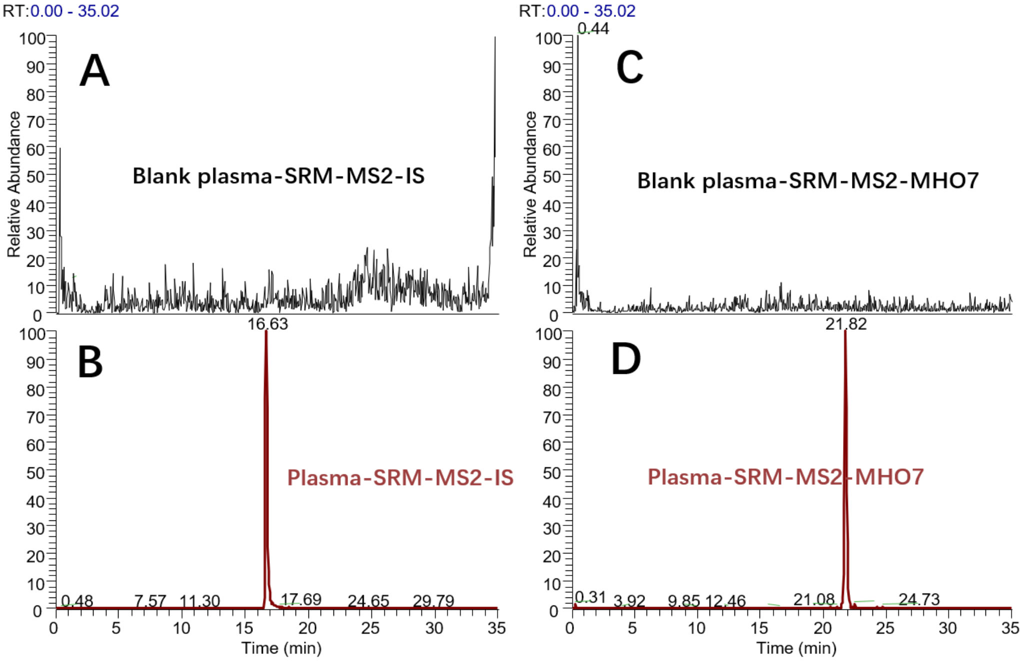

Figure S3. Specificity of MHO7 in plasma. (A) MS2 spectrogram of IS in blank plasma of SRM model. (B) MS2 spectrogram of IS in plasma of SRM model. (C) MS2 spectrogram of MHO7 in blank plasma of SRM model. (D) MS2 spectrogram of MHO7 in plasma of SRM model.

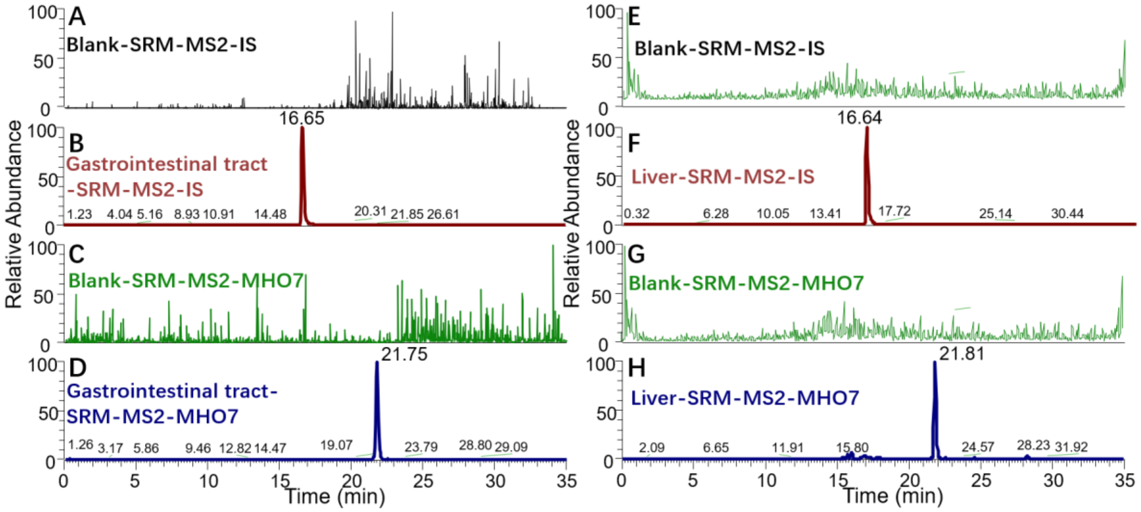

Figure S4. Specificity of MHO7 in tissues. (A) MS2 spectrogram of IS in blank gastrointestinal contents of SRM model. (B) MS2 spectrogram of IS in gastrointestinal contents of SRM model. (C) MS2 spectrogram of MHO7 in blank gastrointestinal contents of SRM model. (D) MS2 spectrogram of MHO7 in gastrointestinal contents of SRM model. (E) MS2 spectrogram of IS in blank liver of SRM model. (F) MS2 spectrogram of IS in liver of SRM model. (G) MS2 spectrogram of MHO7 in blank liver of SRM model. (H) MS2 spectrogram of MHO7 in liver of SRM model.

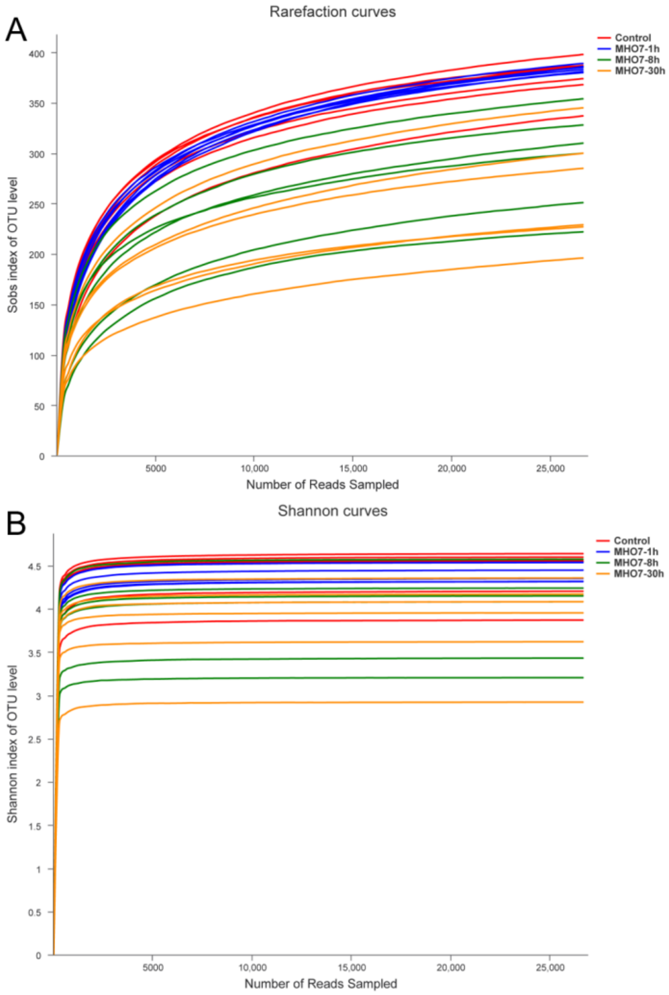

Figure S5. Rarefaction and Shannon index curves of OTU level. (A) Rarefaction curves of OTU level. (B) Shannon curves of OTU level.

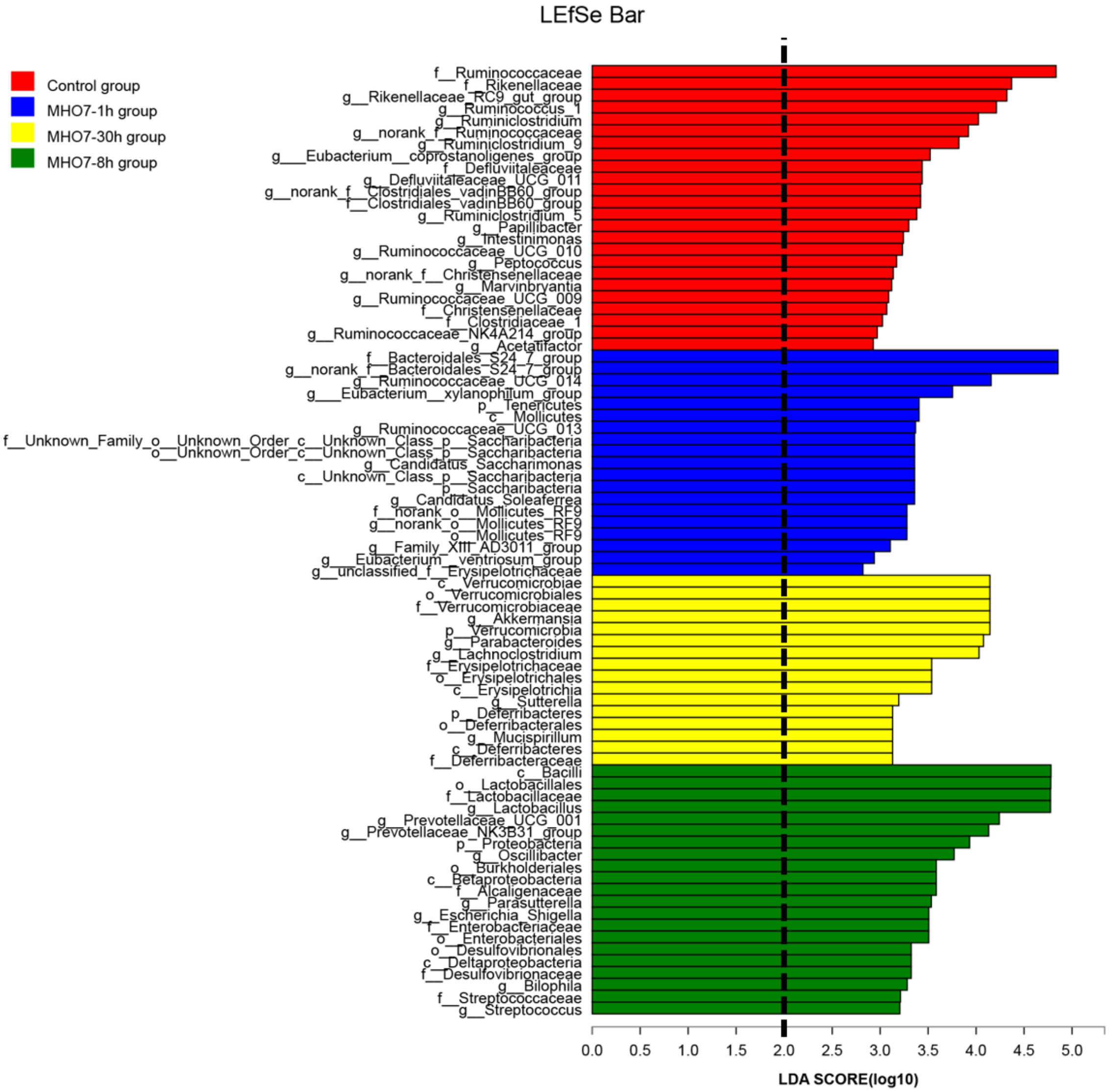

Figure S6. LDA score by Linear Discriminant Analysis (LDA) Effect Size (LefSe) analysis

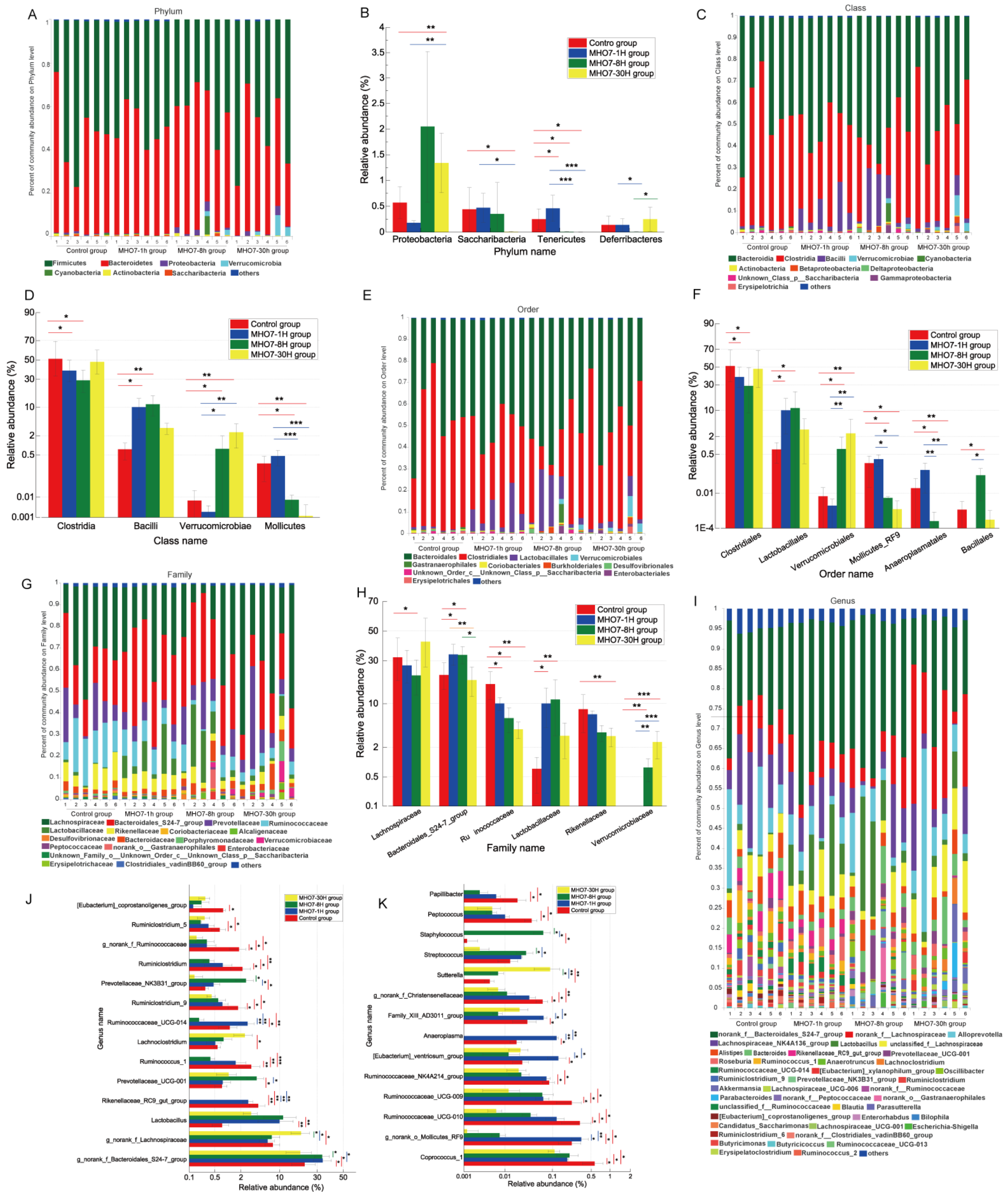

Figure S7. MHO7 modulated the composition of gut microbiota at the different taxonomic levels (n = 6 per group). The distributions of the microbial communities in mice at phylum (A), class (C), order (E), family (G) and genus (I) taxonomic level and the relative abundance of the significant bacterial detected in faecal samples at phylum (B), class (D), order (F), family (H) and genus (J, K) taxonomic level.
