# Supplementary material for: Toxicity, Pharmacokinetics, and Gut Microbiome of Oral Administration of Sesterterpene MHO7 Derived from a Marine Fungus
Source: Mar Drugs. 2019 Nov 26;17(12):667. doi: 10.3390/md17120667 (PMC6950057; doi:10.3390/md17120667)
Supplement: Supplementary file 1 [file supplementary materials-2-annimal.pdf › supplementary materials-2-annimal.pdf]

武汉大学动物实验中心
动物生物安全三级实验室

Wuhan University Center for Animal Experiment/ABSL-III Laboratory

Dear Prof. Hong Kui (洪葵),

The Institutional Animal Care and Use Committee (IACUC) at Center for Animal Experiment, Wuhan University approved your protocol on **Sep. - 25 - 2018**.

Project：The study of antitumor drug MHO7 on pharmacodynamics experiment

AUP Number： 2018106

Approval Date： Sep. - 25 - 2018

Expiration Date： Sep. - 24 - 2019

NOTE: Request for Annual Renewals of ACUPS must be received within the IACUC office 10 days prior to its renewal date.

*Routine Reviews should be submitted at least 6 weeks prior to the scheduled expiration date in order to allow enough time for the review process. Failure to re-submit this AUP for its review will result in automatic closure on the scheduled expiration date.

Although the IACUC office will make every effort to provide 30 day notice regarding required AUP reviews, investigators must satisfy the above requirements.

In the meantime, should you have any questions regarding this notice, please do not hesitate to contact the **IACUC office (027-6875-8757)**.

If you need original approval document, please contact IACUC office (**027-6875-8757**).

Thank you for your cooperation with the IACUC.

Sincerely Yours,

The Institutional Animal Care and Use Committee(Signature)

武汉大学动物实验中心 实验动物管理与使用委员会

湖北省武汉市武昌区东湖路 115 号，430071
115 Donghu Road, Wuchang District, Wuhan, Hubei 430071, P.R.China

电话：(+86)27-68758702
Tel: (+86)27-68758702

传真：(+86)27-68758702
Fax: (+86)27-68758702

武汉大学动物实验中心
动物生物安全三级实验室

Wuhan University Center for Animal Experiment/ABSL-III Laboratory

---

Dear Prof. Hong Kui (洪葵),

The Institutional Animal Care and Use Committee (IACUC) at Center for Animal Experiment, Wuhan University approved your protocol on **Jun. - 04 - 2018**.

Project: The study of antitumor drug MHO7 Pharmacokinetics experiment

AUP Number: 2018058

Approval Date: Jun. - 04 - 2018

Expiration Date: Jun. - 03 - 2019

NOTE: Request for Annual Renewals of ACUPS must be received within the IACUC office 10 days prior to its renewal date.

*Routine Reviews should be submitted at least 6 weeks prior to the scheduled expiration date in order to allow enough time for the review process. Failure to re-submit this AUP for its review will result in automatic closure on the scheduled expiration date.

Although the IACUC office will make every effort to provide 30 day notice regarding required AUP reviews, investigators must satisfy the above requirements.

In the meantime, should you have any questions regarding this notice, please do not hesitate to contact the **IACUC office (027-6875-8757)**.

If you need original approval document, please contact IACUC office **(027-6875-8757)**.

Thank you for your cooperation with the IACUC.

Sincerely Yours,

The Institutional Animal Care and Use Committee(Signature)

武汉大学动物实验中心 实验动物管理与使用委员会

湖北省武汉市武昌区东湖路 115 号，430071
115 Donghu Road, Wuchang District, Wuhan, Hubei 430071, P.R.China

电话: (+86)27-68758702
Tel: (+86)27-68758702

传真: (+86)27-68758702
Fax: (+86)27-68758702
